# Genetic subtypes of type 2 diabetes are distinguished through the lens of abdominal MRI

**DOI:** 10.3389/fgene.2025.1605721

**Published:** 2025-07-16

**Authors:** Elena P. Sorokin, Madeleine Cule, Marjola Thanaj, Nicolas Basty, Brandon Whitcher, Naveed Sattar, E. Louise Thomas, Jimmy D. Bell, Hanieh Yaghootkar

**Affiliations:** ^1^ Calico Life Sciences LLC, South San Francisco, CA, United States; ^2^ Research Center for Optimal Health, School of Life Sciences, University of Westminster, London, United Kingdom; ^3^ School of Cardiovascular and Metabolic Health, University of Glasgow, Glasgow, United Kingdom; ^4^ Joseph Banks Laboratories, School of Natural Science, College of Health and Science, University of Lincoln, Lincoln, United Kingdom

**Keywords:** type 2 diabetes subtypes, magnetic resonance imaging, precision medicine, polygenic risk score, ectopic fat

## Abstract

**Objective:**

This study seeks to understand type 2 diabetes (T2D) heterogeneity through detailed phenotypic characterization of various T2D genetic subtypes using advanced magnetic resonance imaging (MRI) techniques.

**Study design and method:**

MRI data from over 44,000 UK Biobank participants was used to characterize distinct T2D genetic subtypes based on a compendium of imaging-derived phenotypes (IDPs) quantifying body fat distribution, organ volumes, and muscle quality. Partitioned polygenic risk scores (pPS) representing genetic T2D subtypes were associated with adipose tissue distribution across ten compartments, liver and pancreas volume, three muscle mass indices, and fatty acid composition in subcutaneous and visceral fat.

**Results:**

Subtype pPS marked by insulin deficiency were associated with lower subcutaneous fat, while insulin resistance subtypes were associated with higher adiposity with evidence of fat excess in multiple organs, including the pancreas, paraspinal muscle, thigh muscle, iliopsoas muscle, and other organs not routinely quantified at scale in human cohorts. Distinct patterns of muscle mass and fatty acid composition further differentiated subtype pPS, underscoring variation in metabolic profiles linked to specific genetic pathways.

**Conclusion:**

The use of non-invasive MRI to phenotype T2D at a granular level has provided unique insights into the disease’s heterogeneity, confirming and expanding upon known genetic associations. These findings highlight the potential of using MRI for pathophysiological insights into T2D.

## Introduction

Type 2 diabetes (T2D) is a clinically and biologically heterogeneous disease, with wide variation in age of onset, progression, severity of complications, and treatment response. While lifestyle and environmental factors play a substantial role, genetic variation significantly contributes to individual susceptibility and disease presentation. Recent Genome-Wide Association Studies (GWAS) for T2D identified over 600 genetic risk loci that influence clinical symptoms often through cell-type specific genes (Suzuki et al., 2024; Smith et al., 2024; Mahajan et al., 2022; Vujkovic et al., 2020). These and other recent advancements are enabling a more personalized approach for T2D management by integrating clinical features and biomarkers with genomic data to identify and differentiate between diabetes subtypes.

Building on these advances, the T2D Global Genetics Initiative Consortium conducted an extensive GWAS using data from over 2.5 million individuals of diverse ancestries and identified 1,289 variants associated with T2D (Suzuki et al., 2024). Using 37 cardiometabolic phenotypes, these variants were then clustered into eight distinct genetic subtypes. Three of the clusters were linked to β-cell dysfunction – defined by differing relationships with proinsulin (PI), a precursor of insulin that reflects β-cell processing capacity. These included a subtype with elevated proinsulin levels (beta cell +PI), one with low proinsulin (beta cell −PI), and a residual glycaemic cluster with neutral PI levels. These distinctions capture variation in insulin production and secretion capacity, which is central to the development of T2D in individuals whose beta cells fail to maintain glycaemic control, even in the absence of pronounced insulin resistance.

The remaining five clusters were characterised by features of insulin resistance, highlighting the heterogeneity in the ways tissues fail to respond to insulin. These subtypes were labelled according to their predominant phenotype: higher overall body fat, features of metabolic syndrome (e.g., dyslipidaemia, hypertension), obesity, lipodystrophy (impaired peripheral fat storage with ectopic lipid accumulation), and aberrant liver and lipid metabolism. Although traditionally viewed as separate mechanisms, many individuals with T2D present features of both impaired insulin secretion and insulin resistance, and their interplay can influence disease severity and treatment response. These genetic subtypes provided a new framework through which to study T2D, recognising that individuals with the same clinical diagnosis may present markedly different underlying pathophysiological drivers.

Despite these advances, it remains unclear how these genetic subtypes translate into differences in physiological traits measurable *in vivo*. Abdominal magnetic resonance imaging (MRI) offers an opportunity to address this gap by providing precise, non-invasive quantification of body fat distribution, organ size, muscle quality, and fatty acid composition. This study used abdominal MRI acquisitions from over 44,000 individuals in the UK Biobank to measure twenty imaging-derived phenotypes (IDPs) across major tissues and organs. Using deep learning-derived metrics, we examined the relationship between T2D genetic subtypes and organ-specific imaging traits—including intramuscular fat and skeletal muscle mass, internal organ volumes, and ectopic fat depots. Detailed phenotypic profiling using neck-to-knee, non-invasive medical imaging enabled an understanding of how certain genetic configurations may influence the distribution of fat and muscle throughout the human body.

## Methods

### Study overview

20 IDPs were quantified from abdominal MRI scans using deep learning in the UK Biobank imaging cohort (44,646; Table 1) (Littlejohns et al., 2020). These IDPs included measures of subcutaneous adipose tissue (total, abdominal, and thigh), visceral adipose tissue, intramuscular adipose tissue (total, and thigh), liver Proton Density Fat Fraction (PDFF), pancreas PDFF, pancreas volume, vertebral bone marrow PDFF, paraspinal muscle PDFF, total adipose tissue volume, total muscle index, thigh muscle index, and iliopsoas muscle index. Other IDPs included fatty acid composition traits: fractions of monounsaturated fatty acids, polyunsaturated fatty acids, and saturated fatty acids in both abdominal subcutaneous adipose tissue and visceral adipose tissue compartments. Participants who self-identified as a race/ethnicity group with n < 200 total participants (intersected with imaging data) were not included in partitioned polygenic score (pPS) analyses due to a lack of statistical power. Participants with incomplete demographic information or genetic data were excluded. We explored associations between IDPs and cluster-specific pPS in European (n = 37,860), South Asian (n = 452), admixed African (n = 224), and East Asian (n = 207) ancestries as described below.

**TABLE 1:** 20 MRI-derived phenotypes of body composition include adipose tissue depots, muscle quality and indices, and organ volume, in addition to fatty acid composition of adipose tissues.

Phenotype	Description	Unit
Total SAT	Total subcutaneous adipose tissue volume	mL
Abdominal SAT	Abdominal subcutaneous adipose tissue volume	mL
Thigh SAT	Thigh subcutaneous adipose tissue volumes	mL
Visceral adipose tissue	Visceral adipose tissue volume	mL
Liver PDFF	Liver proton density fat fraction	%
Pancreas PDFF	Pancreas proton density fat fraction	%
Pancreas volume	Pancreas volume	mL
Thigh IMAT index	Thigh intramuscular adipose tissue (IMAT) volume, divided by height^2^	index (L/m^2^)
Vertebral bone marrow PDFF	Vertebral bone marrow proton density fat fraction	%
Paraspinal muscle PDFF	Paraspinal muscle proton density fat fraction	%
Internal adipose tissue	Internal adipose tissue volume	mL
Total muscle mass index	Total muscle volume, divided by height^2^	index (L/m^2^)
Thigh muscle mass index	Thigh muscle volume, divided by height^2^	index (L/m^2^)
Iliopsoas muscle mass index	Iliopsoas muscle volume, divided by height^2^	index (L/m^2^)
fSFA (abdominal subcutaneous adipose tissue)	Fraction of fatty acids that are saturated fatty acids in subcutaneous adipose tissue	%
fMUFA (abdominal subcutaneous adipose tissue)	Fraction of fatty acids that are monounsaturated fatty acids in subcutaneous adipose tissue	%
fPUFA (abdominal subcutaneous adipose tissue)	Fraction of fatty acids that are polyunsaturated fatty acids in subcutaneous adipose tissue	%
fSFA (visceral adipose tissue)	Fraction of fatty acids that are saturated fatty acids in visceral adipose tissue	%
fMUFA (visceral adipose tissue)	Fraction of fatty acids that are monounsaturated fatty acids in visceral adipose tissue	%
fPUFA (visceral adipose tissue)	Fraction of fatty acids that are polyunsaturated fatty acids in visceral adipose tissue	%

### Abdominal MRI-derived traits

This study focused on the neck-to-knee Dixon MRI acquisition, the single-slice multi-echo sequences of the pancreas and liver, and the T1-weighted pancreas volume (Littlejohns et al., 2020). Single-slice acquisitions included the Iterative Decomposition of Water and Fat with Echo Assymetry and Least-Squares Estimation (IDEAL) and the gradient echo (GRE). This analysis utilized 44,646 datasets available as of June 2022, athough a total of 100,000 datasets are the ultimate goal of the UK Biobank imaging study. Segmentation of organs from the 3D Dixon volumes was accomplished using a previously published 3D U-Net model (Liu et al., 2021). Briefly, for the liver segmentation, used to quantify PDFF from the single-slice data, a 2D U-Net model was trained on the GRE acquisition. 3D pancreas segmentation was performed on the high-resolution T1w 3D acquisition, which had better contrast and resolution than was available from the Dixon data. A 2D mask was then derived from the 3D pancreas segmentation. Deep learning models were also used to calculate measures of iliopsoas, thigh, and total muscle volume and thigh IMAT (Thanaj et al., 2024a). Muscle volumes and IMAT were indexed to body size by dividing by height squared. Quantification of fatty acid composition of subcutaneous and visceral adipose tissue was measured from the single-slice multi-echo pancreas acquisition (Thanaj et al., 2024b).

Vertebral bone marrow PDFF was derived from the intersection of the quantitative single-slice scan acquired for the liver with the 3D vertebral volume. To segment vertebral bone marrow we developed a deep-learning model trained on manual vertebrae annotations from T1 to S1 on the Dixon MRI data from 120 participants. The model achieved a Dice similarity coefficient of 0.83 on a 20% test dataset. Vertebral bone marrow segmentations were then projected onto the single-slice liver acquisition to define a region of interest for extracting median vertebral bone marrow PDFF. Quality control steps excluded participants with missing or non-intersecting slices (n = 17,462), and those with segmentations covering fewer than 64 voxels (1.9 cm^2^ area; n = 447) (Parkinson et al., 2025).

### Partitioned polygenic scores

We derived partitioned polygenic scores (pPS) to represent each of the eight genetic T2D subtypes identified by the T2D Global Genetics Initiative Consortium (T2D GGI) (Suzuki et al., 2024). This large-scale GWAS, conducted in 2,535,601 individuals of diverse ancestries, identified 1,289 independent loci significantly associated with T2D (p < 5e−8). These loci were grouped into eight mechanistically distinct clusters using unsupervised clustering based on 37 cardiometabolic phenotypes, which included glycaemic and lipid traits, anthropometric and blood pressure measures, biomarkers of liver function, body fat percentage, and imaging-derived phenotypes of visceral adipose tissue, abdominal subcutaneous adipose tissue, gluteofemoral adipose tissue, and liver PDFF (Suzuki et al., 2024). Among these 37 phenotypes, 10.8% (n = 4) were derived from imaging data. Only one (2.7%) of these the phenotypes overlapped with our previous publication, of liver PDFF (Liu et al., 2021), while the remaining three IDPs were reported in another study (Agrawal et al., 2022). A maximum possible sample overlap between the original T2D study and this study is 1.5% of the original study sample or n = 38,312 samples.

The T2D-GGI study found eight distinct T2D genetic subtypes: three subtypes were characterised by β-cell dysfunction (beta cell +PI, beta cell −PI, and residual glycaemic) and five subtypes were associated with different aspects of insulin resistance (obesity, body fat, metabolic syndrome, lipodystrophy, and liver/lipid metabolism). The beta cell +PI and −PI subtypes displayed opposing associations with proinsulin, whereas the residual glycaemic cluster shared some glycaemic traits but was not associated with proinsulin levels. The insulin resistance clusters were defined based on distinct profiles of lipid and anthropometric traits, with the metabolic syndrome cluster also characterised by lower gluteofemoral adipose tissue (Suzuki et al., 2024).

We generated pPSs for each individual in our study cohort by calculating a weighted sum of genotype dosages for variants belonging to each subtype. Specifically, we downloaded the summary statistics from the T2D-GGI study (Suzuki et al., 2024) via the DIAGRAM consortium website (https://www.diagram-consortium.org/) on 1 April 2024 and harmonised variant IDs to dbSNP v140 (https://ftp.ncbi.nlm.nih.gov/snp/). Of the 1,289 significant variants, 1,282 (99.4%) were polymorphic in the UK Biobank imaging cohort (n = 43,491) with imputed genotype data available. Ancestry groups were defined using the UK Biobank’s genetic principal components and reference groupings (https://biobank.ndph.ox.ac.uk/ukb/dset.cgi?id=2442). Field 22006 was provided by the UK Biobank (Bycroft et al., 2018). The pPS for each individual *i* and cluster *k* was calculated using the formula:

$${pPS}_{ik}=\sum_{j=1}^{m}G_{ij\;}\left({c_{jk\;}\circ \beta }_{j}\right)$$

where ${pPS}_{ik}$ is the polygenic score for individual i in each of k clusters in 1≤k≤8 ; c is a binary indicator variable, $c\in \left\{0,1\right\}$ , which indicates cluster membership for each variant j in cluster k as identified in the T2D-GGI study (Suzuki et al., 2024); $\beta_{j}$ is the effect size of variant j in the T2D-GGI GWAS meta-analysis (Suzuki et al., 2024); and $G_{ij}$ is the imputed genotype dosage for individual i at variant j , aligned to the T2D-risk increasing allele. To incorporate effect sizes according to cluster membership, the Hadamard product $\left(c\circ \beta \right)$ was calculated in python using the numpy package, such that the effect sizes of non-cluster containing variants were reduced to zero. pPS calculations using individual-level imputed genotype dosages were then implemented in PLINK1.9 using the --score command, using individual-level genotypes in European (n = 37,860), South Asian (n = 452), admixed African (n = 224), and East Asian ancestries (n = 207) with complete covariates and available imputed genotype data. Initial analysis was performed in the European ancestry group, and replication was performed in non-European ancestry groups.

### Correlation analysis

Correlation analysis was performed in R v.4.2. Out of n = 44,646 participants, n = 22,830 had complete phenotypic information across 20 IDPs, in addition to abdominal SAT and VAT expressed as indices (i.e., divided by height (Smith et al., 2024)). All participants with available phenotype data were included. Pearson correlation coefficients were determined using the *corrplot* package. Correlation between IDPs with BMI adjustment was performed using linear regression (y ∼ x + BMI), where x and y are pairs of IDPs. MRI-derived traits were standardized except for PDFF traits which were rank-normalized.

### Regression modeling

Linear regression was performed in R v.4.2. MRI-derived traits were standardized except for PDFF of liver, pancreas, paraspinal muscle and vertebral bone marrow, which were rank-normalized. MRI-derived traits were regressed on standardized pPS, adjusted for imaging age, biological sex, height, imaging center, T2D status, and the first five principal components (PCs) of genetic ancestry, calculated separately for each ancestry group. For stratified analysis, individuals with BMI ≥ 25 and BMI < 25 were regressed separately. Sex-specific analysis was performed using biological sex (field 31). Sensitivity analysis was performed by additionally adjusting for BMI in both sex-stratified and overall models. For fatty acid fraction traits, blood plasma quantification in the UK Biobank (fields 23454, 23453, 23455) was used as a control, and blood plasma fatty acid fraction regression models were adjusted for age, sex, T2D status, and the first five PCs of genetic ancestry. Z-scores were calculated by dividing standardized β by standard error. Associations were visualized using the *ggplot2* package.

### Estimating genetic differentiation

Fixation index was estimated using Hudson’s method, calculating variance components separately using the ratio-of-averages approach (Bhatia et al., 2013). Fixation index can be used to understand the degree to which populations are genetically distinct from one another, as well as to understand departures from expected heterozygosity at the individual variant level. $F_{ST\;}$ is defined as

$$F_{ST\;}=\frac{H_{T\;}-H_{S}}{H_{T\;}}$$

where $H_{T\;}$ is the expected heterozygosity for the entire population and $H_{S\;}$ is the expected heterozygosity within each population. We estimated fixation index using Hudson’s method within the UK Biobank (n = 6,752 individuals of admixed African ancestry, n = 9,064 South Asians, and n = 2,783 individuals of East Asian ancestry), using return 2442 (https://biobank.ndph.ox.ac.uk/ukb/dset.cgi?id=2442) to define groups. For individual variants, we estimated Hudson’s $F_{ST\;}$ using the following estimator,

$$F_{ST}^{Hudson}=\frac{{\left(p_{1}-p_{2\;}\right)}^{2}-\frac{p_{1}\left(1-p_{1}\right)}{n_{1}-1}-\frac{p_{2}\left(1-p_{2}\right)}{n_{2}-1}}{p_{1}\left(1-p_{2}\right)+p_{2}\left(1-p_{1}\right)}$$

where $p_{i}$ is the estimated allele frequency in group i for $i\in \left\{1,2\right\}$ (2). Fixation indices were calculated for each variant and each pair of populations using 1,254 type 2 diabetes risk variants ascertained in the admixed African ancestry cohort. For clusters of variants, we used a ratio of averages approach, calculating the variance components separately. Leave-one-out resampling was used to estimate the sampling distribution and calculate standard error for each cluster of variants and each pair of populations.

### Permutation testing

To permute the dataset while maintaining the structure between continuous outcomes and covariables such as age and sex, 1,000 random genetic clusters were generated by sampling without replacement from all 1,282 polymorphic variants, using a cluster size of m = 160 variants, the mean size of the original clusters. 1,000 permutation polygenic scores were calculated as described above and regression modeling was performed to simulate the null of no relationship between polygenic score and outcome. Type I error rate was measured as the proportion of times the permutation p-values were as small or smaller than p = 5e−5, the study significance threshold.

## Results

**FIGURE 1:**
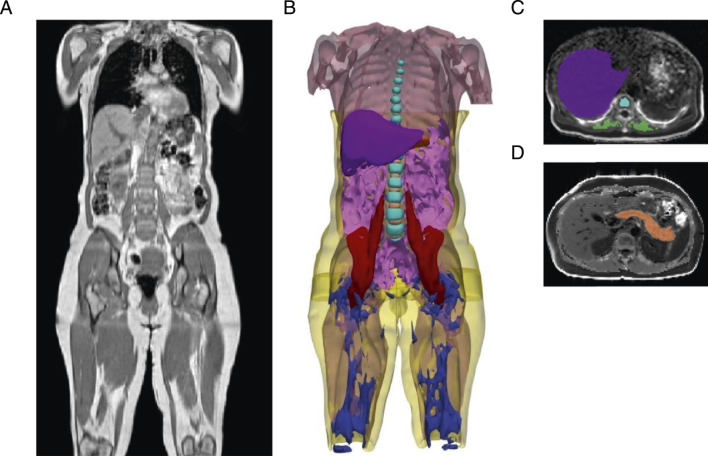
Segmentation of adipose tissue depots, internal organs, and muscles from deep learning of abdominal MRI scans. **(A)** Representative three-dimensional MRI acquisition of the abdomen, extending from the neck to the knee. **(B)** Organs segmented from deep learning models: visceral adipose tissue (magenta), subcutaneous adipose tissue (yellow), liver (purple), pancreas (orange), total muscle (light pink), iliopsoas muscle (red), vertebral bone marrow (cyan blue), intervertebral discs (light orange), thigh intramuscular adipose tissue (IMAT) (dark blue). **(C)** Representative two-dimensional MRI acquisition of the liver (purple) with vertebral bone marrow (cyan blue), and paraspinal muscle (green). **(D)** Representative two-dimensional MRI acquisition of the pancreas (orange).

This study utilizes non-invasive abdominal and neck-to-knee MRI techniques in a population-based cohort to quantify 20 IDPs relevant to type 2 diabetes (n = 44,646). These imaging traits include precise measures of fat in seven anatomical locations (visceral, subcutaneous, liver, pancreas, thigh, paraspinal muscle, and vertebral bone marrow), pancreas volume, skeletal muscle volume and skeletal muscle quality, and fatty acid composition in visceral and abdominal subcutaneous adipose tissues (Figure 1; Table 1). Baseline demographic and clinical characteristics of the study population are summarised in Supplementary Table S1. Pairwise covariance analyses revealed moderate but incomplete correlations across IDPs (Supplementary Figure S1).

**FIGURE 2:**
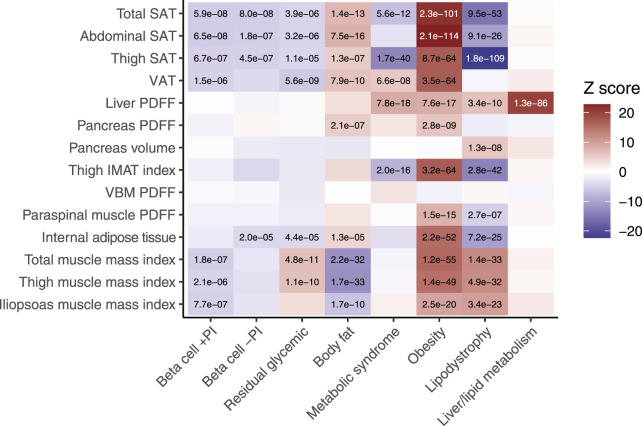
Associations between MRI-derived measures of body composition and eight genetic subtypes of type 2 diabetes. Body fat composition traits derived from neck-to-knee three-dimensional MRI acquisition or quantitative slices of the liver and pancreas. Internal fat is DIXON-derived whole body intramuscular fat. Beta-cell failure clusters include dysfunction with a positive association with proinsulin (beta cell +PI), negative association with proinsulin (beta cell −PI), or neutral (residual glycemic). Insulin resistance clusters include mediation by body fat, obesity, lipodystrophy, metabolic syndrome, and liver/lipid metabolism. The ‘temperature’ of each cell represents the Z-scores (aligned to the type 2 diabetes risk allele) from the standardized effect sizes of the regression model for European participants (n = 37,860). The significance threshold was p < 5e−5, which corresponded to a study-wide estimated type 1 error rate of 5.2% (see *Methods*). Significant associations are labelled with their corresponding p-value. SAT, subcutaneous adipose tissue. VAT, visceral adipose tissue. PDFF, proton density fat fraction. VBM, vertebral bone marrow.

To explore the anatomical differences between genetic subtypes of T2D, we assessed associations between each subtype’s pPS and the MRI-derived traits, stratified by genetic ancestry: European (n = 37,860), South Asian (n = 452), admixed African (n = 224), and East Asian (n = 207). We adopted a significance threshold of p < 5e−5, corresponding to a 5.2% type I error rate based on permutation testing (see *Methods*). In the European cohort, the beta cell +PI pPS was associated with lower muscle mass (e.g., total muscle index, β = −0.016, p = 1.8e−7). In contrast, the residual glycaemic genetic pPS was associated with higher muscle mass indices including of the thigh region (β = 0.021, p = 1.1e−10) (Figure 2; Supplementary Table S2).

Among the insulin resistance subtypes, the body fat pPS and obesity pPS were associated with systemic fat accumulation, including in both subcutaneous and visceral adipose tissues, as well as ectopic fat deposition in the pancreas (p < 5e−5 for all). The obesity pPS was also distinctly associated with higher muscle mass indices (e.g., total muscle: standardized β = 0.050, p = 1.2e−55). The metabolic syndrome pPS was associated with lower thigh subcutaneous adipose tissue (β = −0.058, p = 1.7e−40), higher fat deposition in ectopic sites such as the liver (β = 0.040, p = 7.8e−18) and visceral adipose tissue (β = 0.023, p = 6.6e−8). The lipodystrophy pPS was associated with lower subcutaneous adipose tissue in both the thigh (β = −0.10, p = 1.8e−109) and abdomen (β = −0.050, p = 9.1e−26), higher liver PDFF (β = 0.029, p = 3.4e−1), and higher muscle mass indices (e.g., thigh muscle: β = 0.039, p = 4.9e−32) (Figure 2; Supplementary Table S2).

**FIGURE 3:**
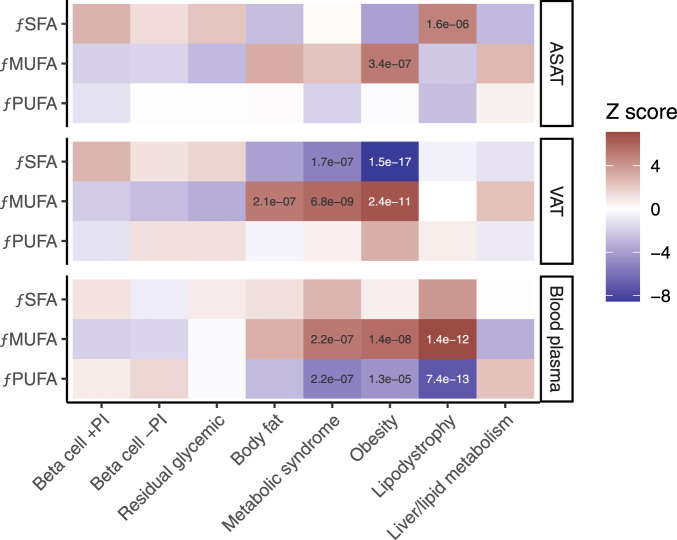
Associations between MRI-derived measures of fatty acid composition and eight genetic subtypes of type 2 diabetes. *f*PUFA, fraction of polyunsaturated fatty acids. *f*MUFA, fraction of monounsaturated fatty acids. *f*SFA, fraction of saturated fatty acids. ASAT, abdominal subcutaneous adipose tissue. VAT, visceral adipose tissue. Blood plasma quantification is shown for comparison. The ‘temperature’ of each cell represents the Z-scores (aligned to the type 2 diabetes risk allele) from the standardized effect sizes of the regression model for European participants (n = 37,860). Significant associations (p < 5e−5) are labelled.

To understand the implications of long-chain fatty acid metabolism for T2D subtype pPS, we explored associations with saturated, monounsaturated and polyunsaturated fatty acids, measured as fraction of total fatty acids, in both visceral adipose tissue and abdominal subcutaneous adipose tissue. Plasma fatty acid fraction is provided, although a limitation of this comparison is that these traits were measured from blood collected at baseline (Julkunen et al., 2023). In the European cohort, both the metabolic syndrome pPS and obesity pPS were associated with higher monounsaturated fatty acids and lower saturated fatty acids in visceral adipose tissue (p < 5e−5; Figure 3; Supplementary Table S3).

Sex-stratified analysis revealed that several associations between subtype pPS and imaging phenotypes were sex-specific. For instance, the association between the metabolic syndrome pPS and visceral adipose tissue was stronger in women (β = 0.041, p = 4.5e−9) (Supplementary Figures S2, S3; Supplementary Table S4). Overall, the findings remained consistent following BMI stratification (Supplementary Figures S4, S5; Supplementary Table S5). To assess the robustness of associations, we conducted sensitivity analyses adjusting for BMI in both overall and sex-stratified models (Supplementary Figures S6–S9; Supplementary Tables S6, S7). Furthermore, associations with abdominal subcutaneous and visceral adipose tissues remained stable when these traits were re-expressed as indices in modified regression models (Supplementary Figure S10; Supplementary Table S8).

**FIGURE 4:**
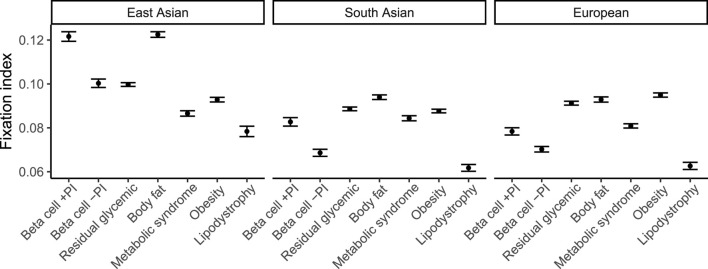
Allelic differentiation across ancestry groups for each cluster of type 2 diabetes variants. Fixation index measured using Hudson’s estimator for pairs of the following populations: European (n = 37,860), South Asian (n = 9,064), and East Asian (n = 2,783) ancestry, in each case using an African ancestry group (n = 6,752) as a common ancestral population. 95% confidence intervals estimated using jackknife resampling are shown.

Few genetic associations replicated in non-European groups, which were all of small sample size of less than 500 participants, such as the association between the liver/lipid metabolism pPS and higher liver PDFF in South Asians (β = 0.22, FDR < 0.05) (Supplementary Table S9). However, ancestry-specific differences in T2D risk profiles have been recently reported (Smith et al., 2024). To develop a theoretical expectation of how well each subtype score might replicate in well-powered non-European cohorts, genetic distance measured as Fixation index (F_ST_) was calculated between large, ancestrally diverse populations including African, South Asian, East Asian, and European ancestries (n > 2,000 for each). In all pairwise comparisons between ancestrally diverse populations, we found the lowest F_ST_ for the lipodystrophy cluster of variants: for example, 0.018 (s.e. 1e−4) for Europeans vs South Asians, 0.051 (s.e 2e−4) between Europeans and East Asians, and 0.063 (3e−4) between Europeans and admixed African ancestry individuals (Figure 4; Supplementary Table S10). For context, these estimates are lower than prior genome-wide F_ST_ estimates, also obtained using Hudson’s estimator, between ancestrally diverse populations (Bhatia et al., 2013). This result suggests that the lipodystrophy pPS may be expected to have higher cross-population prediction accuracy than the other pPS for T2D. While it is well-known that genetic distance from the original study population affects genomic prediction accuracy (Scutari et al., 2016; Martin et al., 2017; Ding et al., 2023), this result raises the question of whether the polygenic score portability problem may concievably extend to risk prediction subtypes, even when all partitioned risk scores are derived from the same GWAS summary statistics.

## Discussion

In this study, we examined the relationships between partitioned polygenic scores (pPS) representing eight genetic subtypes of T2D (Suzuki et al., 2024), and a comprehensive panel of imaging-derived body composition traits. These traits extend beyond traditional anthropometric measures to include regional adiposity (visceral, subcutaneous, hepatic, pancreatic, thigh, paraspinal muscle, and vertebral bone marrow fat), pancreas volume, skeletal muscle size and quality, and fatty acid composition in visceral and abdominal subcutaneous adipose tissues. This comprehensive, quantitative body composition provides insights into the differences between insulin deficiency and insulin resistance pathways and identifies distinctive features of each subtype.

Insulin deficiency subtype pPS, defined by impairments in proinsulin synthesis, processing, or secretion, were consistently associated with reduced subcutaneous fat without compensatory ectopic fat accumulation. The associations of the beta cell +PI pPS with lower muscle mass may reflect poorer overall metabolic health or reduced insulin-mediated muscle accretion during puberty, potentially arising from underlying defects in insulin synthesis or signalling (Sylow et al., 2021). These findings reinforce the notion that insulin deficiency can independently drive T2D development, without necessitating ectopic fat accumulation.

Insulin resistance subtypes—including body fat, obesity, metabolic syndrome, lipodystrophy, and liver/lipid metabolism—were characterised by systemic fat accumulation across multiple depots, with varying patterns of muscle mass. The obesity pPS, for example, was uniquely associated with increased muscle mass, possibly reflecting compensatory hypertrophy or behavioural/lifestyle factors such as physical activity or metabolic processes. This distinction from the body fat pPS supports previous evidence that the biological pathways linking obesity and T2D are heterogeneous (Abraham et al., 2024; Ji et al., 2019; Martin et al., 2021; Martin et al., 2022; Odoemelam et al., 2025; Yaghootkar et al., 2016). The lipodystrophy pPS was marked by low subcutaneous fat and elevated liver PDFF, consistent with the metabolic features of monogenic lipodystrophy (Gonzaga-Jauregui et al., 2020). The liver/lipid metabolism pPS was predominantly associated with increased liver PDFF, signaling a disruption in liver lipid metabolism that is a key feature of metabolic dysfunction-associated steatotic liver disease (Byrne and Targher, 2015). The metabolic syndrome pPS showed a distinct pattern of ectopic fat accumulation (visceral and hepatic) combined with reduced subcutaneous fat, reflecting a redistribution of fat that may contribute to systemic metabolic dysregulation (Shulman, 2014).

Differences in adipose tissue fatty acid composition further distinguished T2D subtype pPS. The link between insulin deficiency subtype pPS and elevated saturated fatty acid fraction in adipose tissues could be due to the role of insulin in regulating fatty acid desaturases, with preclinical studies suggesting insulin deficiency reduces the activity of Δ9, Δ6, and Δ5 desaturases thus leading to a higher level of fatty acid saturation (Vessby et al., 2002). The association of most resistance subtype pPS with higher monounsaturated fatty acids and lower saturated fatty acids in adipose tissues may be explained by the higher stearoyl-CoA desaturase 18:1/18:0 desaturase index reported in subjects with insulin resistance, which would result in a higher fraction of monounsaturated fatty acids (Sjögren et al., 2008; Warensjö et al., 2009). Differences in the ratio of saturated and unsaturated fatty acids could also have implications for relative inflammatory responses across these subtype risk profiles.

This study has some limitations. The lack of robust replication in non-European ancestry groups is likely attributable to limited sample sizes and reduced statistical power. Broader multi-ancestry imaging cohorts are needed to validate these findings. The largest possible sample overlap between the original T2D GWAS meta-analysis and this study corresponds to 1.5% of the original study sample; additionally, the imaging phenotypes examined here contributed minimally (2.7%) to the cardiometabolic traits used to define T2D subtypes in the original study (Suzuki et al., 2024). Although we applied stringent significance thresholds supported by permutation testing to mitigate type I error, potential circularity remains a consideration. Moreover, while MRI provides high-resolution anatomical and compositional data, it does not capture dynamic physiological, cellular, or molecular processes that may further differentiate subtypes. Of relevance, bone marrow fat is estimated to account for roughly 10% of total fat mass in lean adults and this study examines only one marrow fat depot, as opposed to quantifying multiple fat depots as has been done previously (Cawthorn et al., 2014; Xu et al., 2025). Finally, this study employs a population-level analytical framework, which may limit direct clinical translation to individual with T2D without further contextualisation. The mechanisms underlying the associations in this study remain speculative without further experimental validation.

Our findings demonstrate that cohort-based medical imaging can detect subtle and large-scale anatomical and metabolic differences between subtypes of a complex disease such as T2D. This supports the broader application of quantitative imaging for non-invasive disease stratification and highlights its potential to refine our understanding of T2D pathophysiology. This work has implications for the utility of quantitative MRI in the non-invasive, whole-body characterization of complex diseases more broadly and may also inform future strategies to phenotypically profile disease subtypes in response to therapeutic intervention.

## Novelty statement

**What is already known?** Type 2 diabetes (T2D) is heterogeneous, with multiple genetic subtypes and variable phenotypic presentations.

**What this study has found?** Using abdominal MRI acquisitions and 20 quantitative traits from nine organs/tissues in a large population-based cohort, we uncovered distinct differences in fat distribution, muscle quality, pancreas volume, and fatty acid composition across T2D subtype partitioned polygenic scores (pPS).

**What are the implications of the study?** Quantitative imaging can non-invasively delineate subtype-specific profiles in T2D, advancing our understanding of disease heterogeneity and informing personalized management or therapeutic intervention strategies.

## Data Availability

The datasets presented in this study can be found in online repositories. The names of the repository/repositories and accession number(s) can be found below: https://ams.ukbiobank.ac.uk/ams/, UK Biobank (via application).
