# Supplementary material for: Genetic subtypes of type 2 diabetes are distinguished through the lens of abdominal MRI
Source: Front Genet. 2025 Jul 16;16:1605721. doi: 10.3389/fgene.2025.1605721 (PMC12307213; doi:10.3389/fgene.2025.1605721)
Supplement: Supplementary file 1 [file Supplementaryfile1.pdf]

Supplementary Information

Supplementary Figures 2
Supplementary Tables 8

Supplementary Figures

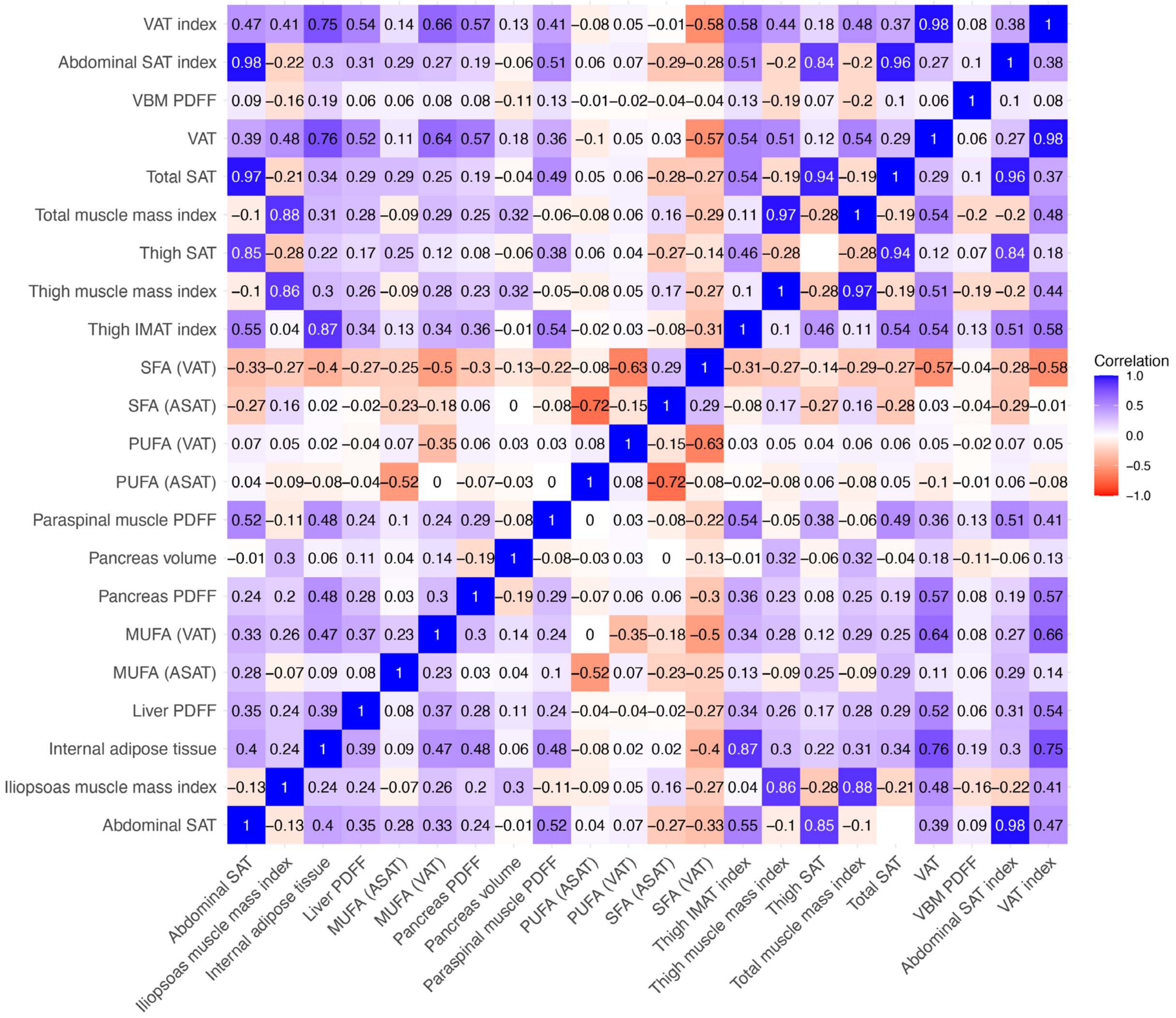

Supplementary Figure 1a. Phenotypic correlations between abdominal MRI-derived traits in 22,830 UK Biobank participants with complete phenotypic data. The color scale reflects the Pearson correlation coefficient which is also displayed on the plot. PDFF, proton density fat fraction. SAT, abdominal subcutaneous adipose tissue. VAT, visceral adipose tissue. Abdominal SAT and VAT, expressed as indices, are included.

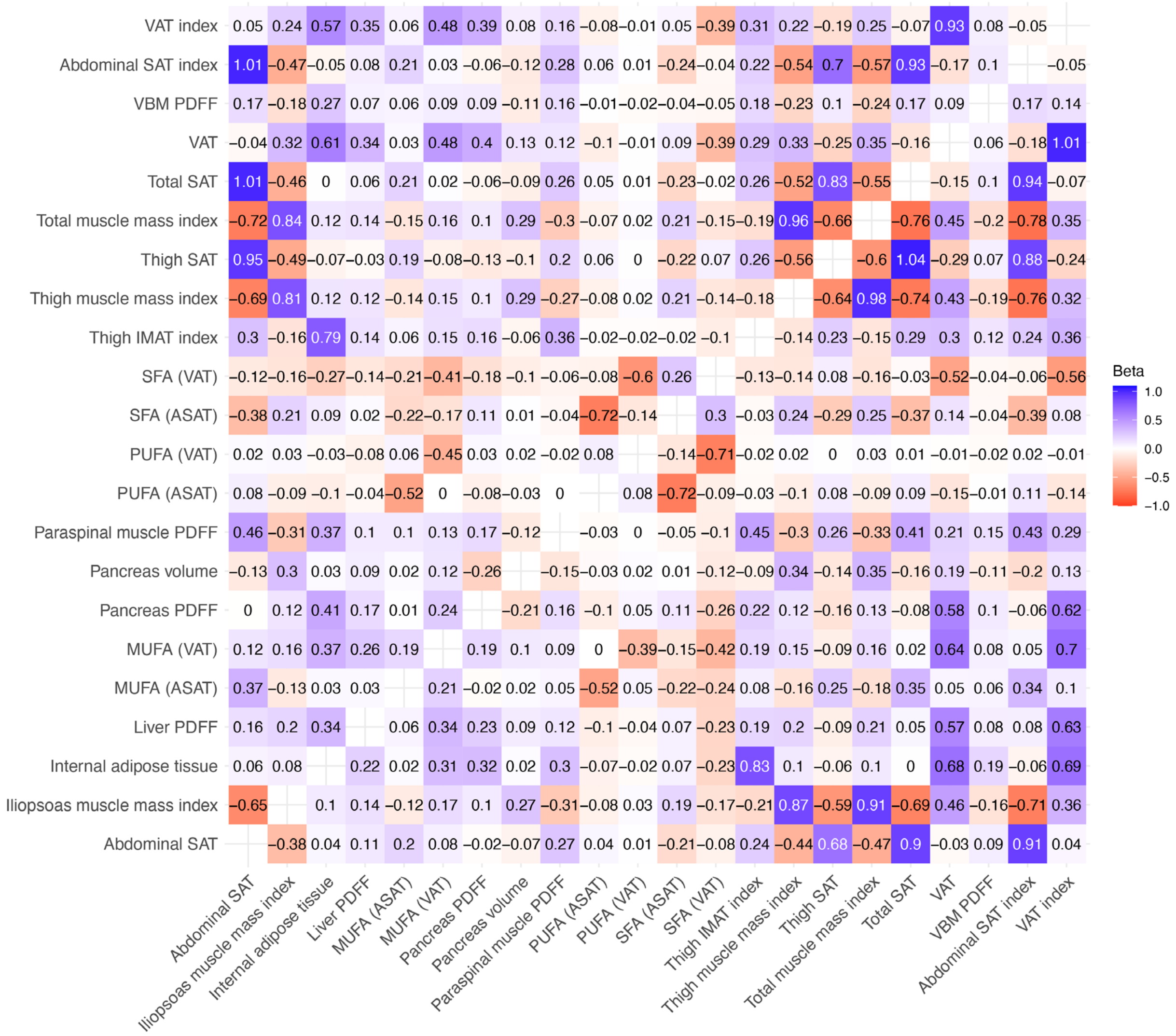

Supplementary Figure 1b. Phenotypic correlations between abdominal MRI-derived traits in 22,830 UK Biobank participants with complete phenotypic data, adjusted for BMI. The color scale reflects the effect size estimate from the regression model, after adjusting for BMI (see Methods). The outcome trait, y, is displayed on the y-axis and the predictor trait is displayed on the x-axis. PDFF, proton density fat fraction. SAT, abdominal subcutaneous adipose tissue. VAT, visceral adipose tissue. Abdominal SAT and VAT, expressed as indices, are included.

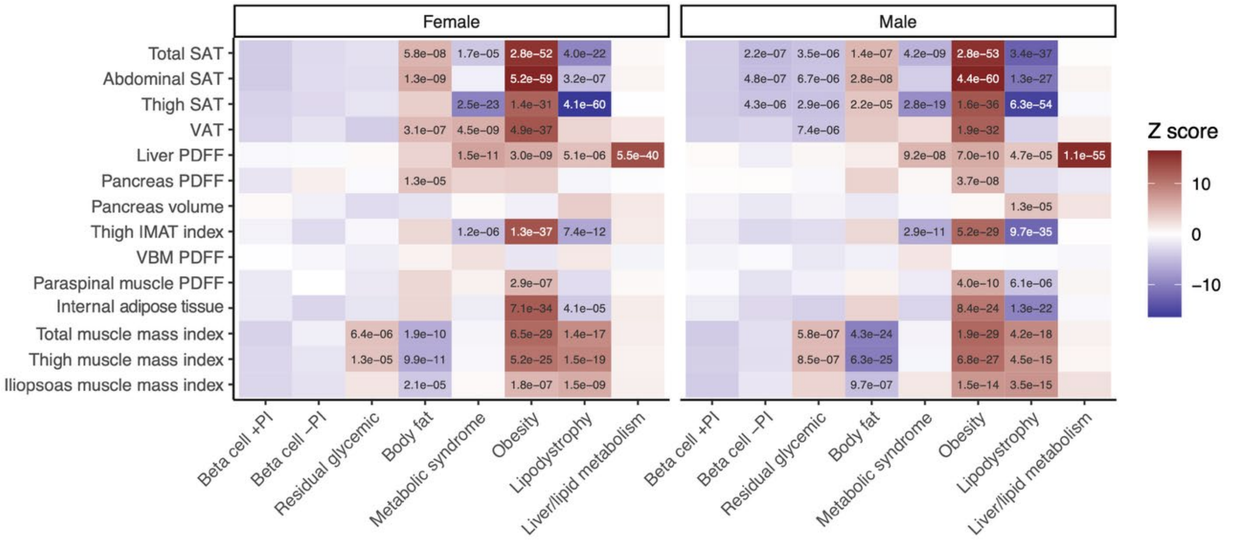

Supplementary Figure 2. Sex-stratified associations between partitioned polygenic scores (pPS) for T2D subtypes and MRI-derived phenotypes reflecting body composition . PI, proinsulin. Significant associations (p<5e-05) are labelled. The ‘temperature’ of each cell represents the Z-scores (aligned to the type 2 diabetes risk allele) from the standardized effect sizes of the regression model for European participants (n=37860). Significant associations (p<5e-05) are labelled.

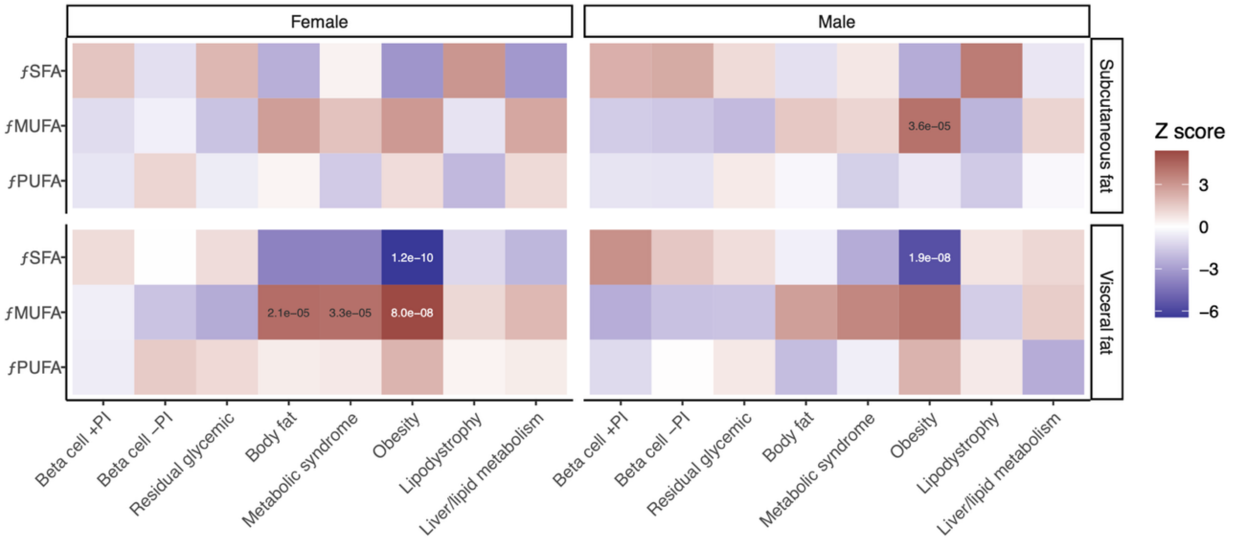

Supplementary Figure 3: Sex-stratified associations between partitioned polygenic scores (pPS) for T2D subtypes and MRI-derived phenotypes reflecting fatty acid composition in adipose tissues: abdominal subcutaneous adipose tissue (subcutaneous fat) and visceral adipose tissue (visceral fat). fSFA, fraction of saturated fatty acids. fMUFA, fraction of monounsaturated fatty acids. fPUFA, fraction of polyunsaturated fatty acids. The ‘temperature’ of each cell represents the Z-scores (aligned to the type 2 diabetes risk allele) from the standardized effect sizes of the regression model for European participants (n=37860). Significant associations (p<5e-05) are labelled.

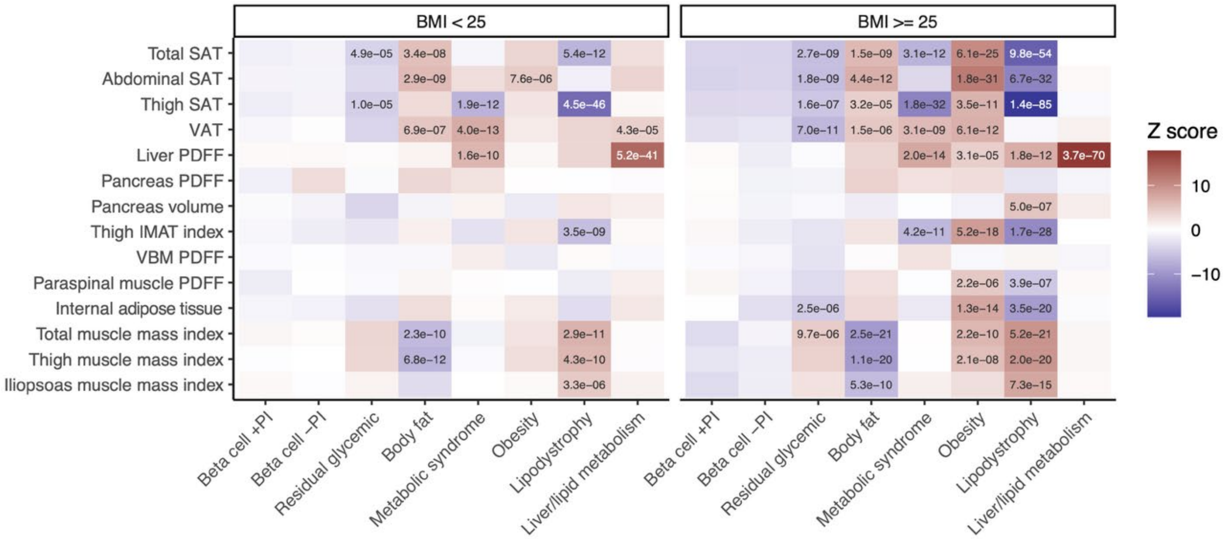

Supplementary Figure 4. Low and high-BMI stratified associations between MRI-derived phenotypes of body composition and genetic subtypes of T2D. The 'temperature' of each cell represents the Z-scores (aligned to the type 2 diabetes risk allele) from the standardized effect sizes of the regression model. Significant associations (p<5e-05) are labelled.

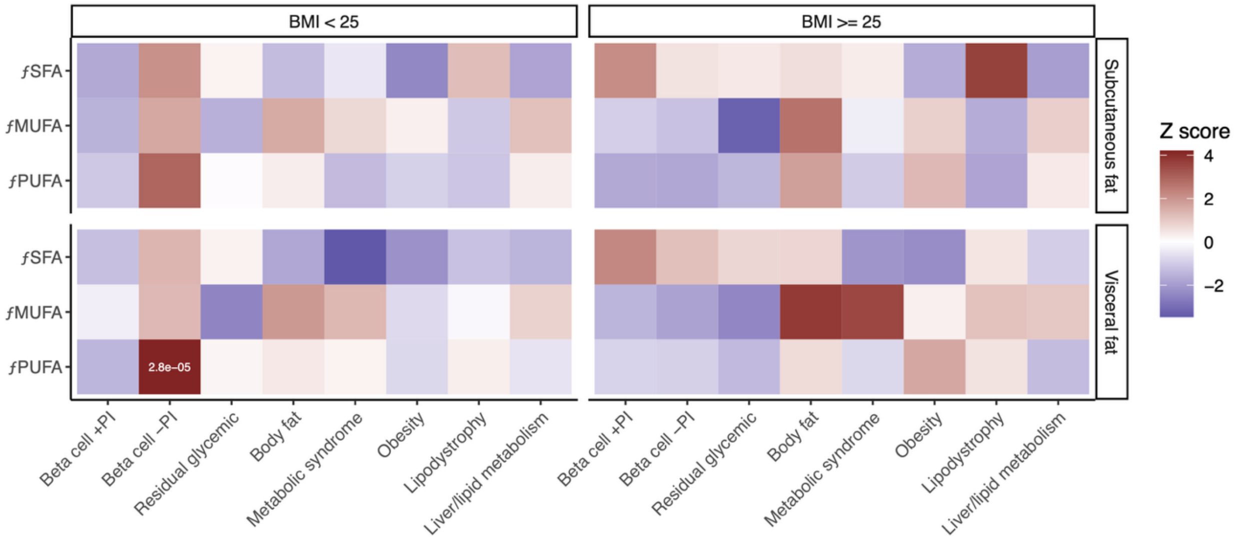

Supplementary Figure 5. Low and high-BMI stratified associations between MRI-derived phenotypes of fatty acid composition in adipose tissues and genetic subtypes of T2D. Significant associations (p<5e-05) are labelled.

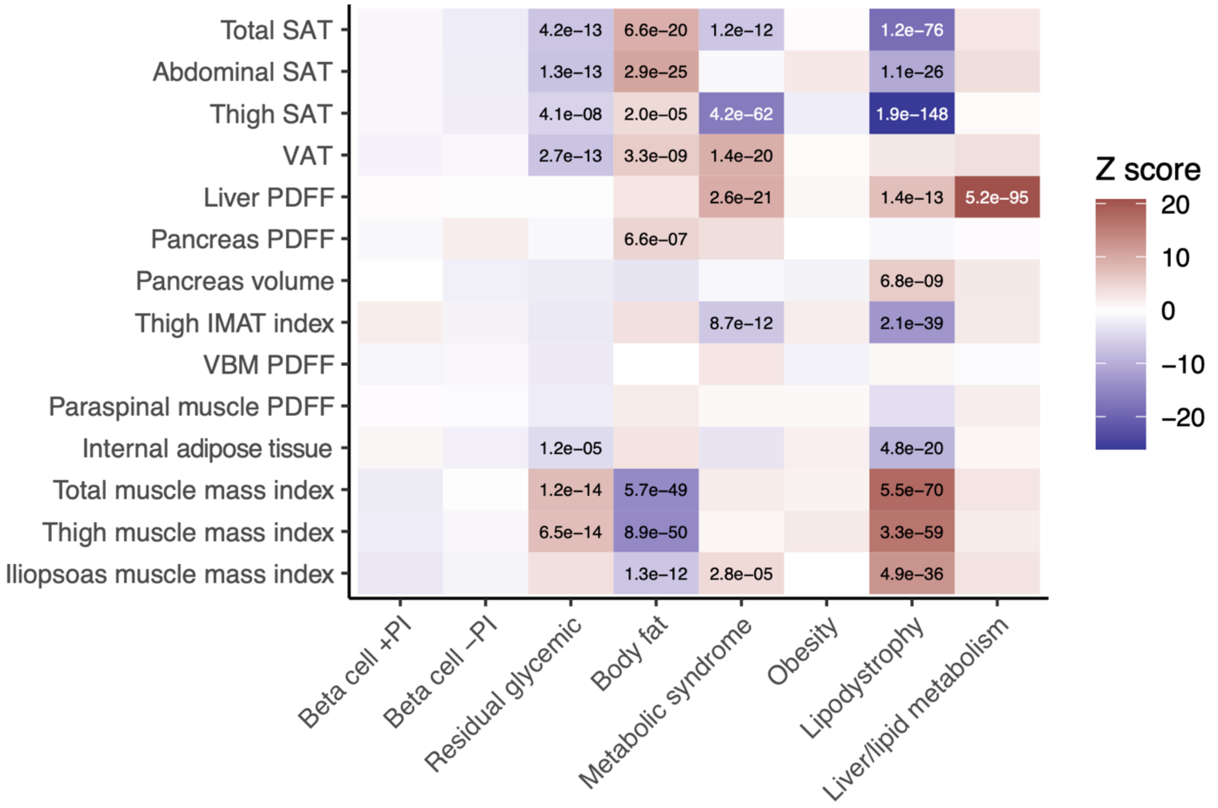

Supplementary Figure 6: Sensitivity analysis was conducted to further adjust for BMI, in addition to base model covariates, for 14 IDPs reflective of adipose tissue compartments. The 'temperature' of each cell represents the Z-scores (aligned to the type 2 diabetes risk alleles) from the standardized effect sizes of the regression model for European participants (n=37860). Significant associations are labelled with the corresponding p-value (significance threshold: p < 5e-05).

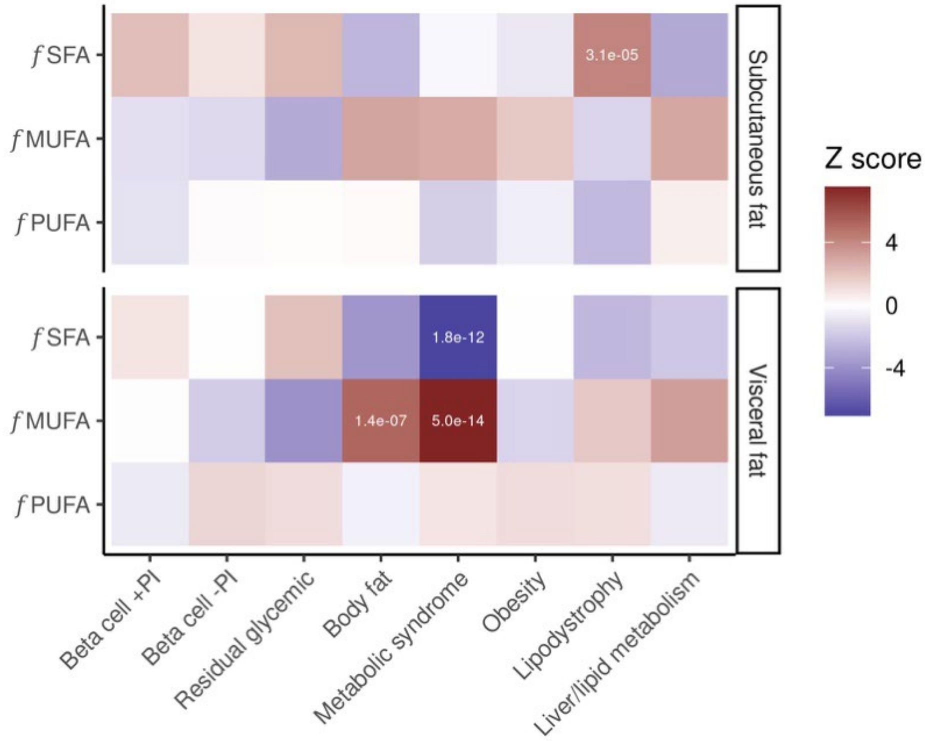

Supplementary Figure 7: Sensitivity analysis, with BMI adjustment in addition to base model covariates, for fatty acid composition of the subcutaneous and visceral fat. fSFA, fraction of saturated fatty acids. fMUFA, fraction of monounsaturated fatty acids. fPUFA, fraction of polyunsaturated fatty acids. The 'temperature' of each cell represents the Z-scores (aligned to the type 2 diabetes risk allele) from the standardized effect sizes of the regression model for European participants (n=37860). Significant associations (p<5e-05) are labelled.

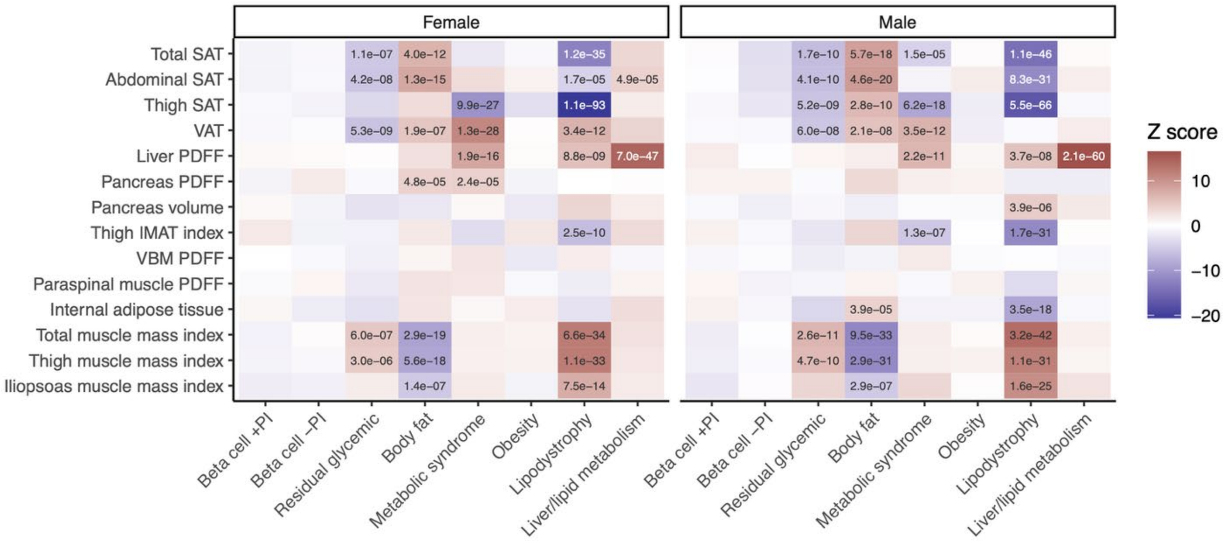

Supplementary Figure 8: Sensitivity analysis for sex-stratified regression models, with BMI adjustment in addition to base model covariates. The 'temperature' of each cell represents the Z-scores (aligned to the type 2 diabetes risk allele) from the standardized effect sizes of the

regression model for European participants (n=37860). Significant associations (p<5e-05) are labelled.

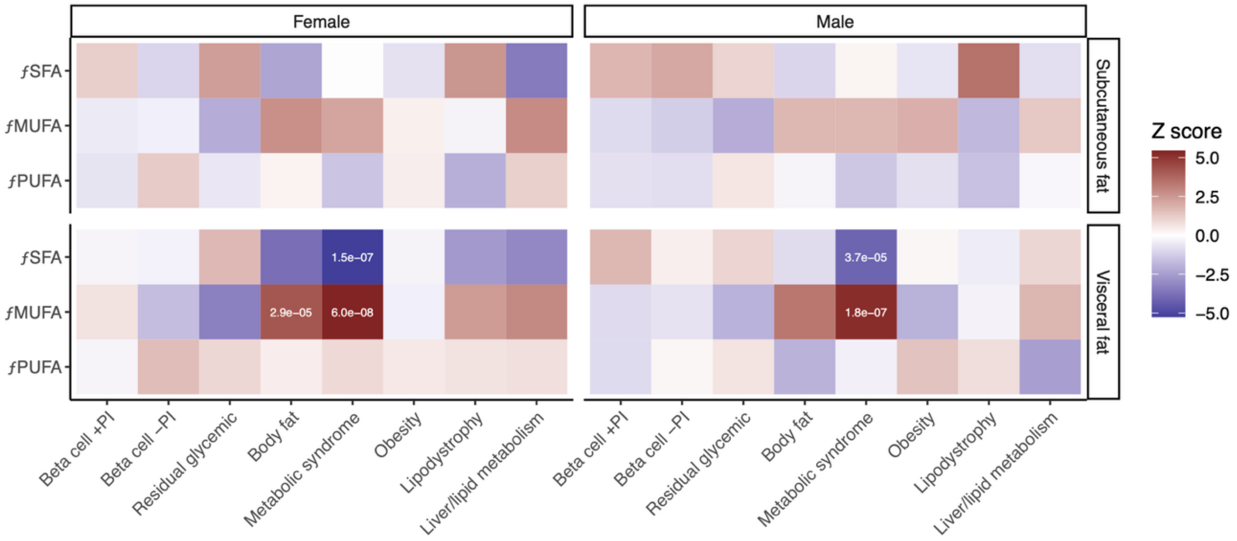

Supplementary Figure 9: Sensitivity analysis for sex-stratified regression models, with BMI adjustment in addition to base model covariates. The 'temperature' of each cell represents the Z-scores (aligned to the type 2 diabetes risk allele) from the standardized effect sizes of the regression model for European participants (n=37860). Significant associations (p<5e-05) are labelled.

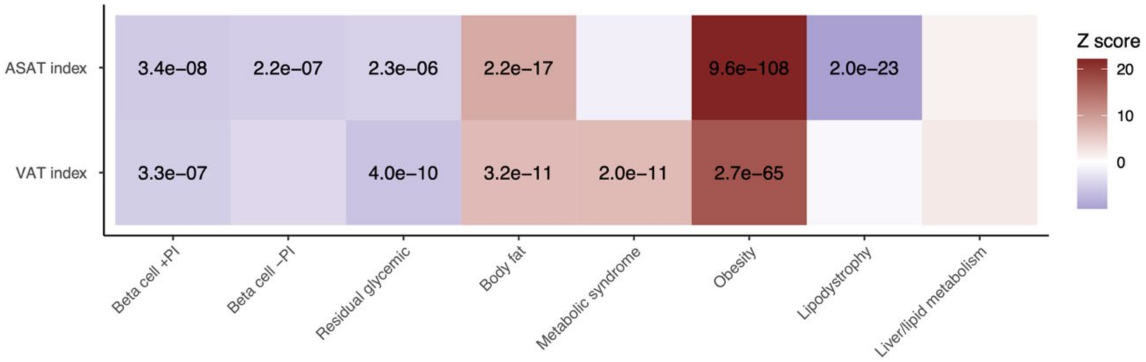

Supplementary Figure 10: Regression of imaging-derived abdominal subcutaneous adipose tissue volume and visceral adipose tissue volume, expressed as indices, on type 2 diabetes subtype partitioned polygenic scores, adjusted for covariates. The 'temperature' of each cell represents the Z-scores (aligned to the type 2 diabetes risk allele) from the standardized effect sizes of the regression model for European participants (n=37860). Significant associations (p<5e-05) are labelled. ASAT, abdominal subcutaneous adipose tissue; VAT, visceral adipose tissue.

Supplementary Tables

Supplementary Table 1: Population demographics for the UK Biobank imaging cohort. PDFF, proton density fat fraction. SAT, subcutaneous adipose tissue. ASAT, abdominal subcutaneous adipose tissue. VAT, visceral adipose tissue. Summary measures are provided as mean (standard error) except for measures of PDFF which are provided as median [interquartile range].

	European		South Asians		Admixed African		East Asians	
	**N**	**mean (se)**	**N**	**mean (se)**	**N**	**mean (se)**	**N**	**mean (se)**
% Female	37680	0.512 (0.0026)	452	0.35 (0.0225)	224	0.567 (0.0332)	207	0.628 (0.0337)
Age at imaging (years)	37680	64.402 (0.0395)	452	61.314 (0.4018)	224	59.08 (0.4908)	207	60.063 (0.5021)
Height (cm)	37680	169.904 (0.0471)	452	166.679 (0.418)	224	168.882 (0.6012)	207	162.488 (0.5344)
BMI (kg/m2)	37680	26.574 (0.0215)	452	26.242 (0.1817)	224	28.5 (0.3151)	207	24.051 (0.237)
T2D prevalence (%)	37680	0.045 (0.0011)	452	0.168 (0.0176)	224	0.143 (0.0234)	207	0.053 (0.0156)
Total SAT (mL)	37860	18260.766 (38.6559)	452	17828.214 (317.3099)	224	20523.895 (651.7979)	207	14549.274 (362.7761)
ASAT (mL)	37860	8455.74 (21.2105)	452	8458.449 (167.0088)	224	9339.018 (339.8319)	207	6505.654 (198.6463)
Thigh SAT (mL)	37860	7459.686 (15.5438)	451	7049.027 (133.1214)	224	8860.752 (276.0793)	207	5767.542 (142.1033)
VAT (mL)	37860	3987.668 (11.9587)	451	3913.385 (83.8392)	224	2656.901 (105.6656)	207	2661.178 (112.1166)
Liver PDFF (%)	30230	2.96 [3.17]	351	3.49 [4.59]	185	2.83 [2.15]	163	2.61 [2.8]
Pancreas PDFF (%)	28657	8.2 [8.99]	343	7.59 [6.34]	163	5.17 [4.5]	158	5.92 [5.51]
Pancreas volume (mL)	36225	58.74 (0.0927)	433	61.632 (0.8469)	210	59.011 (1.6108)	200	58.938 (1.218)
Thigh IMAT index L/m2)	36514	0.27 (6e-04)	434	0.285 (0.0051)	213	0.292 (0.0082)	198	0.203 (0.0054)
VBM PDFF (%)	22838	38.66 [12.54]	251	37.39 [11.51]	136	34.03 [14.6]	107	38.12 [13.59]
Paraspinal muscle PDFF (%)	30244	6.59 [3.94]	351	7.06 [4.65]	185	5.6 [4.15]	163	5.47 [2.52]
Internal adipose tissue (mL)	37860	2559.779 (5.5552)	452	2430.701 (41.5624)	224	2159.322 (60.3967)	207	1889.013 (54.0268)
Total muscle mass index (L/m2)	36516	6.139 (0.0058)	434	6.032 (0.0502)	213	7.088 (0.0959)	198	5.807 (0.0783)
Thigh muscle mass index(L/m2)	36514	2.912 (0.0028)	434	2.9 (0.0246)	213	3.515 (0.0476)	198	2.775 (0.0373)
Iliopsoas muscle mass index(L/m2)	36516	0.221 (2e-04)	434	0.212 (0.002)	213	0.236 (0.0036)	198	0.206 (0.003)
MUFA (ASAT) (%)	28472	0.405 (2e-04)	343	0.417 (0.001)	161	0.409 (0.0025)	158	0.419 (0.0015)
MUFA (VAT) (%)	28483	0.358 (2e-04)	343	0.37 (0.0015)	162	0.343 (0.0029)	158	0.355 (0.0024)

PUFA (ASAT) (%)	28472	0.154 (3e-04)	343	0.171 (0.0016)	161	0.169 (0.0049)	158	0.17 (0.0023)
PUFA (VAT) (%)	28483	0.175 (2e-04)	343	0.195 (0.0021)	162	0.196 (0.0051)	158	0.191 (0.0036)
SFA (ASAT) (%)	28472	0.443 (2e-04)	343	0.413 (0.002)	161	0.424 (0.0036)	158	0.412 (0.0031)
SFA (VAT) (%)	28483	0.472 (2e-04)	343	0.44 (0.0022)	162	0.468 (0.0037)	158	0.459 (0.0031)

Supplementary Tables 2-9 are provided in a supplementary spreadsheet.

Supplementary Table 2: Association study of partitioned polygenic scores (pPS) reflecting eight subtypes of type 2 diabetes (T2D) in a European ancestry subset of the UK Biobank imaging cohort (n=37860). The outcomes were MRI-derived measures of body composition including fat volumes and fat fractions and muscle indices. PDFF, proton density fat fraction.

Supplementary Table 3: Summary statistics for association study of partitioned polygenic scores (pPS) reflecting eight subtypes of type 2 diabetes (T2D) in a European ancestry subset of the UK Biobank imaging cohort (n=37860). Outcomes include MRI-derived measures of fatty acid composition in adipose tissues. VAT, visceral adipose tissue. ASAT, abdominal subcutaneous adipose tissue. SFA, fraction of saturated fatty acids; MUFA, fraction of monounsaturated fatty acids; PUFA, fraction of polyunsaturated fatty acids.

Supplementary Table 4: Sex-stratified association study in the European ancestry subset of the UK Biobank imaging cohort (n=37860).

Supplementary Table 5: Association study of MRI-derived measures of body composition and fatty acid fraction, stratified by high BMI (>=25) or low BMI (< 25) in the European ancestry subset of the UK Biobank imaging cohort (n=37860).

Supplementary Table 6: Sensitivity analysis for association study of 20 MRI-derived traits and partitioned polygenic scores (pPS) for T2D subtypes. In addition to base model covariates, BMI is added as an additional covariate.

Supplementary Table 7: Sensitivity analysis for sex-stratified association study of 20 MRI-derived traits and partitioned polygenic scores (pPS) for T2D subtypes. In addition to base model covariates, BMI is added as an additional covariate.

Supplementary Table 8: Association study of abdominal subcutaneous adipose tissue (ASAT) and visceral adipose tissue (VAT) with pPS for T2D subtypes. ASAT and VAT are expressed as indices (divided by $height^2$).

Supplementary Table 9: Ancestry-stratified association study of 20 MRI-derived traits reflecting body composition and fatty acid composition in non-European groups in the UK Biobank imaging cohort: South Asians (n=451), admixed African ancestry (n=224), and East Asians (n=207).

Supplementary Table 10: **Fixation index quantifies allelic differentiation across ancestry groups for each cluster of type 2 diabetes variants.** F_{ST} statistic measured using Hudson's estimator for pairs of the following populations: European (n=37,860), South Asian (n=9064), and East Asian (n=2783) ancestry, in each case using an African ancestry group (n=6752) as a common ancestral population. 95% confidence intervals were estimated using jackknife resampling (see Supplementary Methods).

Cluster	European-admixed African	European-South Asian	European-East Asian	Admixed African-East Asian	Admixed African-South Asian
Beta cell +PI	0.078 (0.00013)	0.018 (3.1e-05)	0.065 (0.00011)	0.122 (0.00017)	0.083 (0.00015)
Beta cell -PI	0.07 (0.00013)	0.022 (5.7e-05)	0.06 (1e-04)	0.1 (0.00017)	0.069 (0.00013)
Residual glycemic	0.091 (1.6e-05)	0.022 (5.2e-06)	0.067 (1.3e-05)	0.1 (1.7e-05)	0.089 (1.5e-05)
Body fat	0.093 (3e-05)	0.022 (8e-06)	0.069 (2.3e-05)	0.123 (3.2e-05)	0.094 (2.7e-05)
Metabolic syndrome	0.081 (4.9e-05)	0.026 (1.7e-05)	0.06 (4e-05)	0.087 (5.8e-05)	0.084 (5.2e-05)
Obesity	0.095 (3.2e-05)	0.023 (1e-05)	0.068 (2.7e-05)	0.093 (3.3e-05)	0.088 (2.9e-05)
Lipodystrophy	0.063 (0.00027)	0.018 (1e-04)	0.051 (0.00023)	0.078 (0.00035)	0.062 (0.00028)
